# Contextual Modulation of Binary Decisions in Dyadic Social Interactions

**DOI:** 10.3389/fnbeh.2021.715030

**Published:** 2021-08-26

**Authors:** Thorsten Fehr, Anja Achtziger

**Affiliations:** ^1^Department of Neuropsychology, Center for Cognitive Sciences, University of Bremen, Bremen, Germany; ^2^Center for Advanced Imaging, Universities of Bremen and Magdeburg, Bremen, Germany; ^3^Department of Political and Social Sciences, Zeppelin University, Friedrichshafen, Germany

**Keywords:** decision-making, social interaction, multiple attributes, prosocial behavior, self-defense, reactive aggression, quasi-realistic design

## Abstract

The present experimental design allowed binary decisions (i.e., to choose between proactive approaching or withdrawing behavior). These decisions were made on complex social interaction scenarios displayed on videos. The videos were taken from a first-person perspective. They were preceded by one sentence each that provided additional information about the context of the displayed scenario. The sentence preceding the video and the video jointly provided a context of emotional valence. That context varied from trial to trial. We observed that provocative and threatening videos produced predominantly fear and anger responses. Fear was associated with withdrawal decisions, while anger led to approach decisions. Negative contextual information increased the probability of approach decisions in aggressive provocative videos; positive contextual information enhanced the chance of approach decisions in socially positive videos. In neutral situations, displayed in videos, the probability of the approach behavior was reduced in case of negative contextual information. Yet, the probability for approach behavior was increased if positive contextual information preceded neutral videos. Our experimental setup provided a paradigm that can be adapted and accommodated for the examination of future research questions on social decisions in multidimensional, complex social situations.

## Introduction

Social interaction, especially in conflict situations, is characterized by ambiguity and uncertainty (e.g., Ramirez and Andreu, 2006). The interpretation of an action is based on the assessment of the situation, in which the action is shown, the interaction partners in this situation, and the personal learning history that is related to this or similar contexts as well (Anderson and Bushman, 2002). Thus, how a person interprets a specific social interaction like the clench of someone's fist can reach from victory to violence (= destructive aggression). Further, this interpretation of an interaction is often modulated by both the individual learning history and the properties of the context, in which the interaction is embedded.

Whether a given context impacts behavior in an appropriate and socially accepted way is often determined by social norms that are learned in an individual's lifelong socialization (Anderson and Bushman, 2002; Wahl, 2009; Fehr, 2012). Sometimes, however, people are confronted with ambiguous situations that cannot be easily interpreted. For example, in a self-defense situation, fighting as a reactive aggressive act is acceptable in most societies and cultures. In these highly dynamic interactions, the situation itself and its implications based on an individual's learning history might play the most important role for choices of actions depending on the current context. One idea is that context-related activation of specific memory networks that contain experience-based perception-action scripts or stereotypes of behaviors (e.g., Fehr et al., 2014) suggests possible actions considered as adequate in this context (Anderson et al., 1998; Bushman, 1998; Anderson and Bushman, 2002).

It was discussed that the contextual modulation of social choices might operate at least on two hypothetical levels. First, a context activates related memory scripts (Anderson et al., 1998; Fehr et al., 2014), and second, these memory scripts potentially modulate attention by top-down processes (Ramirez and Andreu, 2006; Dominguez-Borras et al., 2008).

### Decision Processes in Aversive-Provocative and/or Threatening Social Situations

Two strategies of coping with highly aversive social situations have been prominently discussed—flight and fight behavior (e.g., Cannon, 1929; Berkowitz, 1993). Aversive episodes are described as physically arousing for an individual (Arun, 2004). At the emotional level, fight tendencies have been discussed along with anger, and flight tendencies with fear (Lück et al., 2005). The affective valence of a situation is assumed to be activated subliminally and pre-attentively rather than consciously processed. This activation of the affective valence is claimed as one important aspect in decision processes in critical social situations (cf., Cacioppo et al., 1993; Slovic et al., 2005). In general, it is postulated that quick responses to situations occur whenever the link between this situation and behavior is overlearned (e.g., Anderson and Bushman, 2002). Note that an automatic response on a stimulus or situation cannot be expected to new and unfamiliar situations. The reason is that there are either no associations with a certain behavior in this case, or only weak ones [see Todorov and Bargh (2002)]. In unfamiliar situations, most researchers would assume that controlled processes of reasoning dominate cognition in order to decide what behavior could be adequate in this situation. The question arises, if forced choices between two options on how to respond to a certain social situation are affected by information provided prior to this situation. These choices should be especially difficult in social situations, for which an individual does not have an automated response immediately available. A classical situation could be being threatened by a person with a knife. This situation is luckily unfamiliar for most of us, and therefore, we cannot respond to it automatically in case it would arise.

In the present study, we intend to answer this question with a quasi-realistic experimental design. For this purpose, we manipulated two kinds of information. The first kind of information was presented in one sentence that constituted a specific social context (e.g., “Frank is known to be a very friendly man”). This information established a context for a social interaction that was afterward presented in a short video clip. Each social interaction displayed in the video clips was filmed from a first-person perspective. The videos showed the beginning of a social interaction between a spectator (i.e., the participants) and another person that was not known by the participants. Participants were asked to decide how they would respond in this situation. In particular, participants were asked if they would decide to get proactively involved in the social interaction (e.g., by beating the assaulter), or if they would prefer to withdraw calmly from the social interaction (see *The Present Study* and *Sentences, Video-Clips, and Personality Traits*, for details).

### Potential Enhancement of the Likelihood of Reactive Aggressive Decisions

A meta-analytic approach by Bettencourt et al. (2006) claims that a provocation increases the likelihood of aggressive actions [see also, Bettencourt and Miller (1996)]. In accordance with current aggression theories like the General Aggression Model (GAM; Anderson and Bushman, 2002), the contextual modulation of behavior is explicitly described as a key mechanism in the genesis of aggressive behavior. Previous research that explored information preceding aggressive stimuli as driving factors behind decisions on whether or not to show aggressive behavior used pictographical stimuli such as pictures, videos, verbal stimuli, or even haptic sensation (e.g., Berkowitz and LePage, 1967; Carver and Ganellen, 1983; Anderson et al., 1998; Verona and Curtin, 2006; Coyne et al., 2012). Instead of these pictographical stimuli, we displayed short sentences prior to social interactions shown in video-clips and expected that these sentences modulated participants' behavioral responses on the videos. We claimed that these sentences would modulate the responses as they would be processed as an additional source of information (= attribute 1 in each experimental trial). The video-clip was referred to as attribute 2 in the experimental decision trials.

### The Present Study

Our experimental design differed from many other experimental approaches that were previously used in order to activate aggression-related social emotions. Some of these previous experimental designs use imagery/recall of angry or violently threatening situations (Dougherty et al., 1999; Kimbrell et al., 1999; Damasio et al., 2000; Pietrini et al., 2000) to induce fear and/or anger. Other studies run well-elaborated, classic laboratory tasks like the Taylor Aggression Paradigm (Taylor, 1967) to induce revenge-like aggression. We presented short video-clips that displayed complex social interactions in terms of highly realistic scenarios [see Fehr et al. (2014)]. In our paradigm, participants got actively involved in the social interactions that they watched in the video-clips by choosing one out of two behavioral options. Participants could either decide to proactively approach the situation (e.g., by pushing back an aggressive offender in a reactive aggressive context or shaking hands in a socially positive context) or withdraw from the social interaction (i.e., to remain calm and passive).

As mentioned above, right before each video-clip, a sentence occurred on the computer screen (e.g., “Martin is known to be a violent guy”) that created the context we expected to influence participants' decisions in the social interactions. Hence, we combined two sources of information (i.e., context attributes) about the social interaction in each experimental trial. The first source was the sentence (= contextual attribute 1). The second source of information was the video-clip (contextual attribute 2) presented in each trial.

All social interactions were filmed in everyday life contexts as for instance in public places in a city, in underground car parks, in gardens and parks, and so forth. The video-clips displayed social interactions that were either a threat (i.e., an unknown person attacks or provokes the participant physically) or socially positive (i.e., an unknown person encounters the participant proactively and friendly) or the social interaction was rather neutral (e.g., an unknown person is present, but sends neither threat nor prosocial cues). We predicted that emotional information on a social context that precedes a social interaction (i.e., sentences prior to the video-clips) modulates decisions distinct in different kinds of social interaction scenarios (i.e., shown in video-clips). Based on our past experience and current theoretical models we propose the following predictions:

(1) Evaluation of the sentences (= contextual attribute 1): Analogously to Fehr et al. (2014), we expected that sentences preceding the video-clips with violent, affectively negative contents will be rated as more arousing than sentences with socially positive content and more than sentences with a neutral content. Arousal ratings will be higher for sentences with socially positive content than for neutral sentences.
  Valence ratings of the sentences will be lowest for sentences with negative content, moderate for sentences with neutral contents, and highest for sentences with a positive content (Bradley and Lang, 1994).
  For the ratings of how intense participants experienced a sentences, we expected that ratings would be higher for negative sentences than for positive and for neutral sentences. Further, we predicted that these intensity ratings would be higher for positive sentences than for neutral sentences (cf., Bradley and Lang, 1994). As participants were also requested to categorize each sentences in one of four emotional categories (i.e., anger, fear, serenity, and happiness), we proposed a prediction on these categorizations as well. Specifically, we expected that the emotional categorization of the sentences would end up in a bimodal categorization for sentences with negative contents. For sentences with a negative content, we predicted that they would be mostly categorized as anger or provoking [see Ramirez and Andreu (2006)]. Sentences with neutral contents would be most often categorized as generating feelings of serenity, while sentences with a socially positive content would be categorized as provoking feelings of happiness (cf., Fehr et al., 2014).
(2) Evaluation of the social interactions displayed in video-clips (= attribute 2): Based on the findings by Fehr et al. (2014), we expected that videos showing provocative aggressive interactions would be rated as more arousing than videos with positive social interactions, as more arousing than videos with neutral social interactions. The latter videos were claimed to be less arousing than videos with positive social interactions.
  Based on their own life experience, participants were expected to be most familiar with socially positive and neutral interactions compared to aggressive provocative social interactions resulting in higher familiarity ratings. Unambiguity ratings for the contents of the video-clips (i.e., the question to what extent the social interaction shown in the videos can be recognized) were expected to be higher rated than a moderate level of 5 on a 10-point rating-scale for all categories of the video-clips. This means that the interpretation of the social interactions was expected to be rather clear in all three categories of social interactions.
  We assumed that the categorization of the video-clips based on the affect they induce would result in the same profile for negative (here, aggressive provocative videos bimodally rated as anger and fear inducing), socially positive (predominantly rated as inducing happy feelings), and neutral (producing serenity) social interactions as for the sentences [see predictions 1 above, and Fehr et al. (2014)].
  In the video evaluation part, participants were asked to decide whether they would approach, remain passive, or withdraw in the given situation. According to Fehr et al. (2014), we expected that in aggressive social interactions, participants would mostly decide to either withdraw from the social interactions or remain passive. In socially positive interactions, we expected that the participants would prefer to approach, and in the neutral situations, we expected that participants would mostly decide to approach or remain passive.
(3) Expected interactions between sentences and video-clips in the experimental run: Sentences preceding the video-clips (i.e., the social interactions) were expected to strengthen behavioral tendencies associated with a certain kind of social interaction. For example, sentences providing negative social information like “Thomas is known to harm weaker persons” were maintained to support approach tendencies in response to provocative aggressive social interactions (Bettencourt and Miller, 1996; Bettencourt et al., 2006). The impact of sentences with positive and negative social information on decisions in neutral social interactions displayed in the video-clips was explored. In addition, we scrutinized behavioral tendencies when sentences with socially positive contents (e.g., “Simon always helps people in trouble”) preceded video-clips with positive social interactions (e.g., being greeted by another person).
(4) Correlation analyses: We expected that traits potentially related to physical aggression [see Hampel and Selg (1975) and Buss and Perry (1992)] correlate with proactive approach tendencies in provocative aggressive contexts (Ramirez and Andreu, 2006). Furthermore, for provocative aggressive interactions (video-clips), anger ratings obtained in the stimulus evaluation part will correlate positively with approach tendencies and fear ratings will correlate positively with withdrawal tendencies (cf., Strüber and Fehr, 2009).
(5) Following suggestions by Schmitt et al. (2016), who explicitly asked for a broader exploration of trait-related behavioral tendencies, we planned several exploratory data analyses. For instance, relationships between the decision on how to respond to social interactions (presented in the video-clips) and a couple of trait measurements [e.g., personality and several aggression-related scores; see Hampel and Selg (1975) and Buss and Perry (1992)] were investigated.

## Methods and Procedures

### Participants

The sample consisted of 30 university students (18 females). Age ranged from 19 to 28 years (mean age 22.9 ± 3.0). Participants self-reported no psychiatric disorders or being in psychotherapeutic treatment and were all mother-tongue German speakers. All participants were familiarized with the stimulus presentation (i.e., the sentences, followed by video-clips, and how to use the response buttons when choosing an option of how to act in the social interaction displayed in the video-clips). Participants were informed about the study's procedure and gave written and informed consent to participate. The experimental setup was designed according to the Code of Ethics of the World Medical Association (Declaration of Helsinki, published in the British Medical Journal, July 18, 1964).

### Sentences, Video-Clips, and Personality Traits

**Figure 1:**
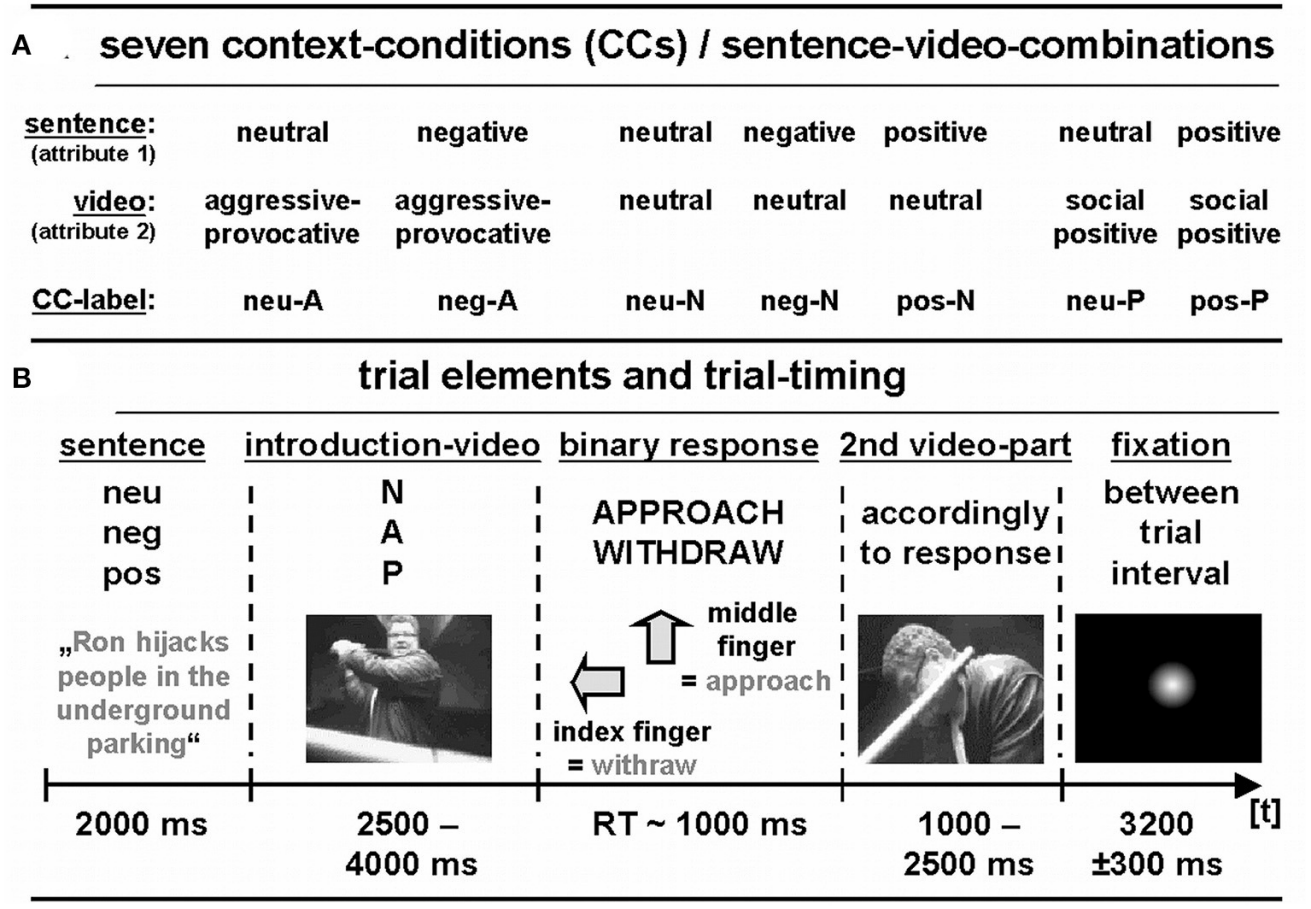
**(A)** Emotional context conditions (CCs). **(B)** Trial elements and trial timing.

The video-clip inventory used in the present study was a further development of a previously used inventory introduced by Fehr et al. (2014) labeled as FNVAId (*F*irst-person *N*aturalistic *V*ideo *A*ffect *I*nventory *d*ecision). The FNVAId inventory is a computer- and video-based instrument developed to investigate decisions in quasi-realistic, complex social interactions. Participants are asked to decide in a forced-choice task, if they like to get actively involved in an affectively laden (negative or positive) or neutral social interaction. Each video-clip begins with a short introductory scene that freezes between 2,500 and 4,000 ms. Alternatively, participants can decide to withdraw from the social interaction (see Figure 1B for illustration of the sequence of trial elements), what we would interpret as participants' intention to remain passive in this interaction. After the decision is made, or after a maximum of 2,000 ms passed without any decision, the remaining part of the social interaction was displayed and presented the consequences of the participant's decision. This remaining part of the social interaction lasts between 1,000 and 2,500 ms. The video-clip shows a realistic decision-related continuation of the social interaction. For instance, after a participant decided to withdraw, the video-clip showed the withdrawal, interruption, or slowing down of the displayed action. In case the participant decided to get actively involved in the social interaction, the video showed approach behavior (e.g., beating the assaulter in case of a self-defense scenario or shaking hands in a socially positive interaction context).

All video-clips presented dyadic interactions filmed from a first-person perspective. The social interactions were either of a Neutral (N), provocative Aggressive (A), or socially positive (P) nature. We displayed 20 different video-clips per each of these three categories of social interactions two times in two experimental runs. The video-clips (= attribute 2) were preceded by German sentences that provided social information that could potentially be used as information suggesting the nature of the subsequent social interaction's context that was displayed in the video-clips (= attribute 1). These sentences provided information that was either negative (i.e., aggressive or threatening such as “Tom just stole your mothers handbag.”), positive (i.e., friendly such as “It's always a pleasure to meet with Simon.”), or neutral (i.e., neither of a negative social nature nor of a positive social nature such as “Andreas walks in the park watching birds.”). Video-clips displaying aggressive-provocative interactions were preceded by sentences with emotionally neutral or negative social content, positive video-clips were headed by sentences with emotionally neutral or positive social content. Neutral video-clips were preceded by sentences with negative, positive, or neutral social content.

Thus, there were seven different *combinations* of sentences and video-clips (= context conditions = CCs, see Figure 1A for an overview). For each of the seven CCs, there were 20 different versions presented in a first and a second run each (thus, 40 trials per CC category in all). Showing each CC twice per participants allowed us to compute an estimation of response consistency. That is to test if participants responded to a particular CC trial in the same manner (e.g., chose to withdraw both times when the same CC trial was presented again). The CCs were presented in a nonstationary-probabilistic, pseudo-randomized sequence (Friston, 2000) across the two experimental runs (each run consisted of 140 trials, i.e., 140 decisions). The run order was balanced across participants.

After making decisions on how to respond on a social interaction displayed in 280 trials in two experimental runs, participants evaluated each video through a computer-based in-house software developed for the purpose of rating videos. Further, each participant assessed each of the sentences on arousal on an 11-point scale (ranging between lowest arousal = 0 and highest arousal = 10; paper-pencil procedure). In addition, each participant categorized the emotion that s/he experienced in response to a social interaction shown in a video-clip into one of the following four categories: anger, fear, happiness, or serenity. The sentences were rated additionally on valence on an 11-point scale (most negative valence = −5; most positive valence = 5), and intensity on an 11-point scale (low intensity = 0; high intensity = 10; cf. Bradley and Lang, 1994). Video-clips were rated additionally on familiarity on an 11-point scale (lowest = 0; highest familiarity = 10), unambiguity on an 11-point scale (lowest = 0; highest unambiguity = 10). The video-clips were categorized regarding the following behavioral options: approach, remain passive, and withdrawal [see Fehr et al. (2014)].

After these post-experimental assessments (i.e., stimulus evaluation) of the sentences and the video-clips, participants completed the following questionnaires: (1) the “Fragebogen zur Erfassung von Aggressivitätsfaktoren (Questionnaire for the assessment of aggression factors/traits)” (FAF), by Hampel and Selg (1975). This questionnaire covers the dimensions spontaneous (proactive) aggression, reactive aggression, impulsiveness, auto-aggression, and inhibition. (2) We administered the “Aggression Questionnaire” (AQ) by Buss and Perry (1992) that measures tendencies to anger and verbal and physical aggression. (3) Participants completed the “Sechs Faktoren Test (Six Factor Test, SFT)” by Von Zerssen (1994); see also Drieling et al. (2007), and finally, they rated their personality on (4) the NEO-FFI, by Costa and McCrae (1992); German version by Borkenau and Ostendorf (1993).

### Statistical Analyses

Several repeated measurement ANOVAs were calculated to justify subsequent statistical analyses on pairwise comparisons of different context conditions (= CCs, see Figure 1A, and above). Significant *post-hoc* tests were only reported and illustrated when they were significant according to Bonferroni-Holm-adjusted *p*-values to control for effects of multiple comparisons.

To analyze the decisions, a preference index (PI) was calculated. It indicated the probability of choosing to approach an interaction (i.e., to participate actively in the social interaction) relative to the sum of all choices (i.e., the sum of all choices to approach and of all choices to remain passive/or to withdraw from the interaction). This relative probability was computed separately for each of the seven context conditions (CC). The scores of the PI ranged between 0 (= a participant chose to remain passive or to withdraw in each of her/his decisions) and 1 (= a participant chose to approach in each of her/his decisions). Differences in the PI between CCs were analyzed with 2 (within: neutral vs. emotional SENTENCE) × 2 (within: neutral vs. emotional VIDEOS) repeated-measurement ANOVAs. These ANOVAs were computed separately for the provocative Aggressive (A) and the socially positive (P), and the Neutral (N) interactions displayed in the video-clips.

As each combination of a specific sentence with a specific video-clip (i.e., a particular trial) was identically presented two times per participant (see above), we could analyze how consistent the decisions were in the different CCs. For the purpose of having an indicator of decision consistency, we calculated an Uncertainty Index (UI). The UI was computed separately for each CC as the ratio between the number of all decisions that were inconsistently made for a specific combination of a sentences and a video-clip and all decisions made in this CC. Thus, a UI score of 0 meant that all decision were consistent, while a UI score of 1 indicated that all decisions were inconsistent. As for the PI (see above), differences in the UI between CCs were analyzed with 2 (within: neutral vs. emotional SENTENCE) × 2 (within: neutral vs. emotional VIDEOS) repeated-measurement ANOVAs.

## Results

### Assessments of the Sentences and Video-Clips (Stimulus Evaluation)

#### Sentences (Evaluation of Context Attribute 1)

**Figure 2:**
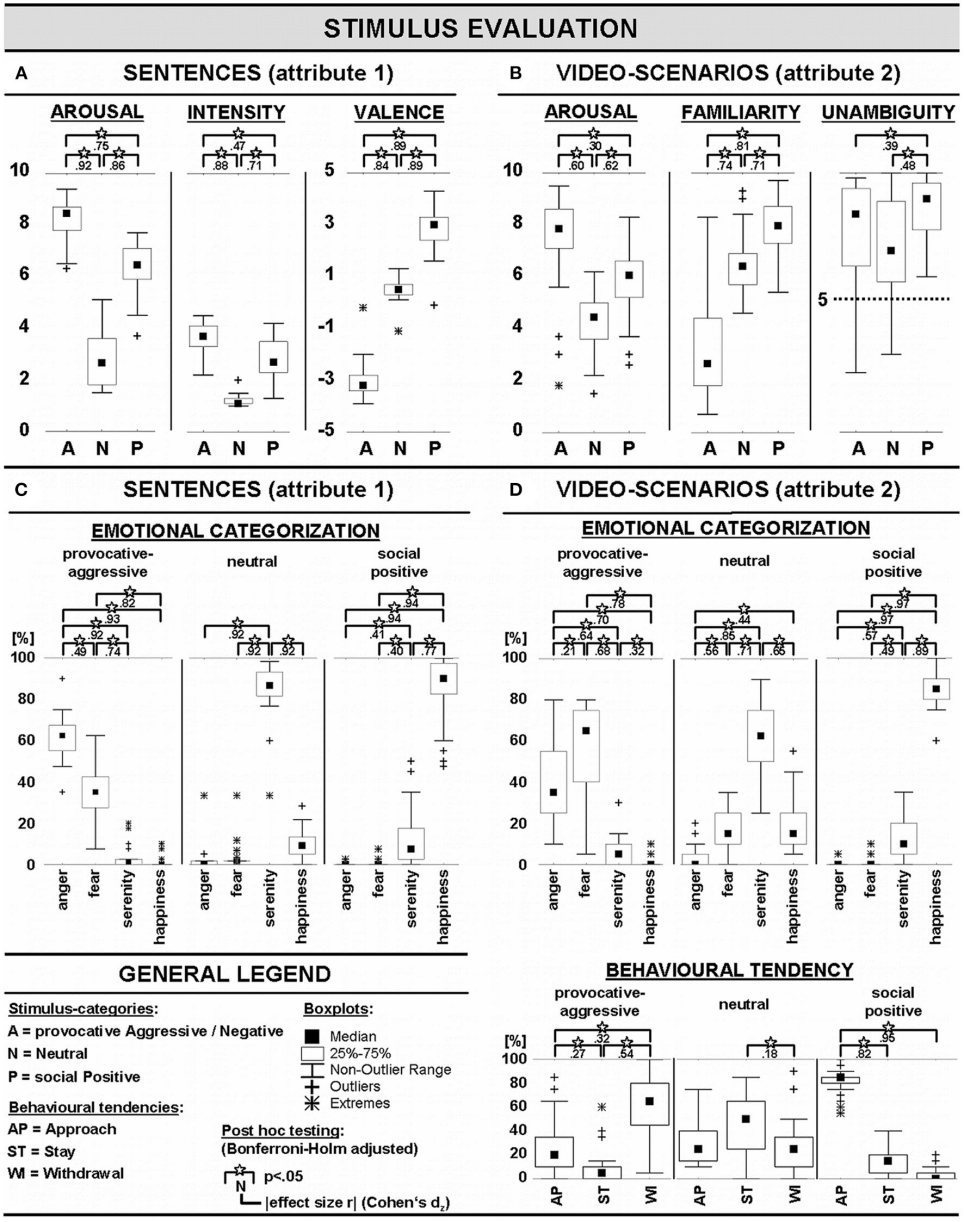
Evaluation of sentence- and video-stimuli (**A,C**: sentences, **B,D**: videos).

Details about the assessments of the sentences preceding the video-clips are illustrated in Figures 2A,C. There were main effects for AROUSAL [*F*_(2,58)_ = 416.9; *p* < 0.001, GG-Epsilon = 0.87] and INTENSITY (cf. Bradley and Lang, 1994) [*F*_(2,58)_ = 170.1; *p* < 0.001, GG-Epsilon = 0.95]. These main effects were explained by high arousal and intensity ratings of sentences with negative social information (i.e., aggressive provocative content), followed by sentences describing positive social information (socially positive content), and lowest arousal and intensity ratings of sentences with neutral information (neutral content).

A main effect of VALENCE [*F*_(2, 58)_ = 384.8; *p* < 0.001, GG-Epsilon = 0.58] was explained by significantly higher negative ratings for negative sentences (aggressive provocative social content), ratings around 0 for neutral sentences, and rather positive ratings for positive sentences (socially positive content). There was a significant interaction between the emotional categories of SENTENCEs (levels: neutral, negative, and positive social content) and the CATEGORIZATION of the emotions triggered by these sentences into the four categories of anger, fear, serenity, and happiness, *F*_(6, 174)_ = 540.8; *p* < 0.001, GG-Epsilon = 0.49. The interaction could be explained by the fact that emotions generated by negative sentences were mostly categorized as anger or fear, emotions triggered by neutral sentences were mostly categorized as serenity, and emotions caused by positive sentences (socially positive) were mostly categorized as feelings of happiness (see Figure 2C for details).

#### Video-Clips (Evaluation of Context Attribute 2)

Details about the assessments of the video-clips (i.e., the social interactions) on post-experimental stimulus evaluation are illustrated in Figures 2B,D. There were main effects of AROUSAL [*F*_(2, 58)_ = 40.1; *p* < 0.001, GG-Epsilon = 0.65], FAMILIARITY [*F*_(2, 58)_ = 184.3; *p* < 0.001, GG-Epsilon = 0.63], and UNAMBIGUITY [*F*_(2, 58)_ = 18.7; *p* < 0.001, GG-Epsilon = 0.96]. Provocative aggressive video-clips received the highest AROUSAL ratings, followed by socially positive video-clips, and neutral video-clips. Socially positive video-clips were rated as most FAMILIAR, followed by neutral video-clips and provocative aggressive video-clips. UNAMBIGUITY ratings were highest for socially positive video-clips.

There was an interaction between the CATEGORY of the social interactions presented in the video-clips (within: neutral, provocative aggressive, and socially positive interactions) and the CATEGORIZATION of emotions provoked by the video-clips (within: anger, fear, serenity, and happiness) [*F*_(6, 174)_ = 271.9; *p* < 0.001, GG-Epsilon = 0.46]. This interaction could be explained by high ratings of anger and fear as responses on provocative aggressive interactions, by high ratings of serenity for neutral social interactions, and by high ratings of happiness for socially positive interactions (see Figure 2D, upper panel for details).

There was an interaction between CATEGORY of the video-clips (negative, positive, and neutral) and BEHAVIORAL TENDENCY on how to respond in this social interaction [within: approach, remain passive, and withdraw; *F*_(4, 116)_ = 82.3; *p* < 0.001, GG-Epsilon = 0.63]. This interaction was due to a high frequency of withdrawal choices, followed by a lower frequency of approach choices in provocative aggressive interactions. For neutral interactions, participants chose predominantly to remain passive. In socially positive interactions, participants most often picked the option to approach this social interaction (for more details see Figure 2D lower panel).

### Experimental Runs: Modulatory Effects on Decisions and Uncertainty in Different Context Conditions (CC)

The preference index (PI; see above) was calculated separately for each of the seven context conditions (CCs). Note that a score of 1 on the PI indicated that a participant chose to approach a social interaction in 100% of a specific CC (e.g., in socially positive interactions). A score of 0 on this index implied that a participant chose never to approach a social interaction, but preferred to remain passive or to withdraw (see *Statistical Analyses* for further details). In a first step, we calculated a repeated-measurement ANOVA including PIs of all seven CCs as a justification for all subsequent statistical analyses: *F*_(6, 174)_ = 101.2; *p* < 0.001, GG-Epsilon = 0.51.

**Figure 3:**
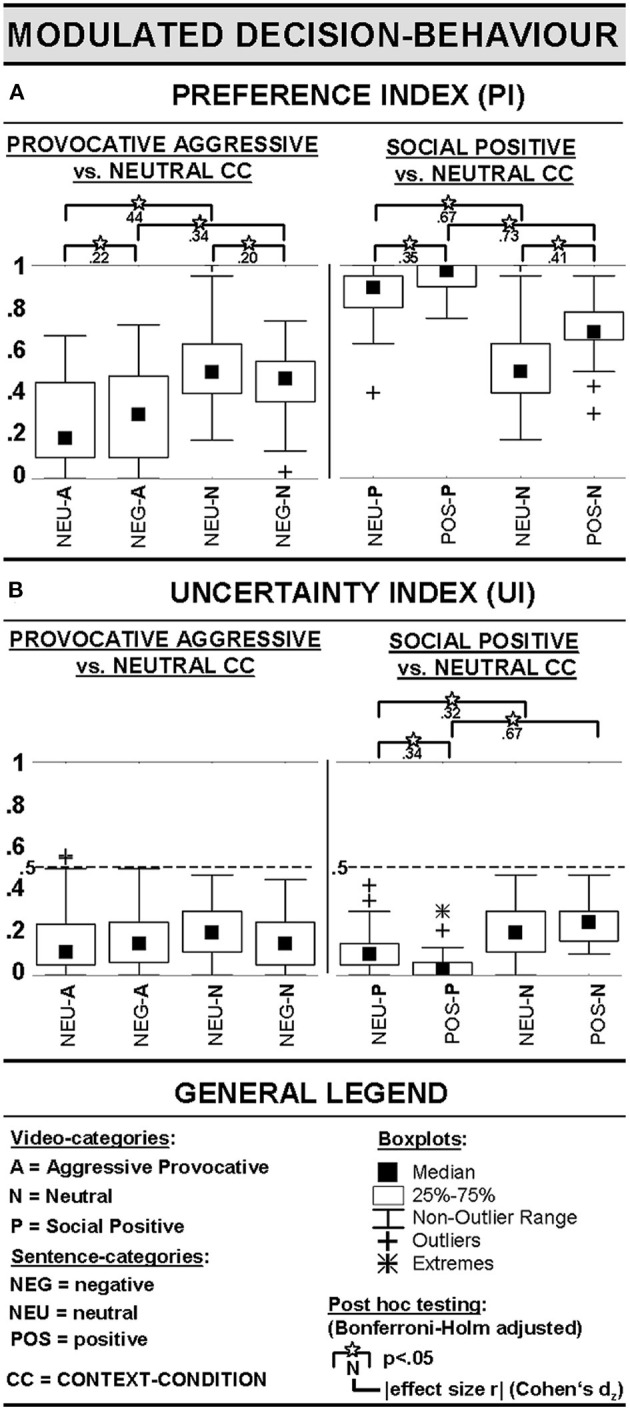
Preference Index (PI, **A**) and Uncertainty Index (UI, **B**).

An ANOVA that tested neutral (NEU) and negative (NEG) sentences (= context attribute 1) as a subset of sentences as one within-factor and the subset provocative aggressive (A) and neutral (N) video-clips (= context attribute 2) as a second within-factor revealed an interaction between SENTENCES (within: NEU vs. NEG) and CATEGORY of the video-clips (within: A vs. N) [*F*_(1, 29)_ = 12.8; *p* < 0.01, GG-Epsilon = 1]. There was also a main effect of the factor CATEGORY of the video-clips [*F*_(1, 29)_ = 28.0; *p* < 0.001, GG-Epsilon = 1]. Interactions and/or main effects could be explained by generally lower scores on the preference index (PI) in CCs that included provocative aggressive video-clips. Furthermore, provocative aggressive video-clips that were preceded by negative sentences compared to neutral sentences resulted in higher scores on the PI, while neutral interactions (i.e., video-clips) preceded by negative sentences compared to neutral video-clips preceded by neutral sentences ended up in lower PI scores (for details see Figure 3A, left panel).

Including neutral (NEU) and positive (POS) sentences (= context attribute 1) as one within-factor and socially positive (P) and neutral (N) video-clips (= context attribute 2) as a second within-factor in an ANOVA revealed an interaction between SENTENCE (within: NEU vs. POS) and CATEGORY of video-clips (within: P and N) [*F*_(1, 29)_ = 5.5; *p* < 0.05, GG-Epsilon = 1], a main effect of SENTENCE [*F*_(1, 29)_ = 32.2; *p* < 0.001, GG-Epsilon = 1], and a main effect of CATEGORY of video-clip [*F*_(1, 29)_ = 158.3; *p* < 0.001, GG-Epsilon = 1]. Interactions and/or main effects were based on higher PI scores in positive social interactions (i.e., video-clips) compared to the neutral interactions presented in video-clips and on higher PI scores whenever video-clips, irrespective of their emotional content, were preceded by sentences with positive information (for details see Figure 3A, right panel).

The uncertainty index (UI) estimated the consistency of participants' decisions in identical combinations of sentences and video-clips (see above). To justify subsequent statistical analyses, we calculated a repeated-measurement ANOVA including UIs of all seven CCs. This ANOVA turned out as significant, *F*_(6, 174)_ = 11.5; *p* < 0.001, GG-Epsilon = 0.75. All UIs were significantly below 0.5 when tested in one-tailed *t*-tests (*p* < 0.05; for details see Figure 3B). This finding underlined that our participants did not ignore the information provided in the sentences prior to the video-clips, but processed this information. Otherwise, testing the UIs against 0.5 would not have become significant as participants would have randomly picked option of how to respond to an interaction. Interactions in UIs between CCs were not of further interest in the present study, and therefore not computed.

### Relationships Between Decisions, Self-Assessments of Sentences and Video-Clips, and Personality Traits

All reported correlations in this section were significant (i.e., the respective *T*-tests showed significance levels of *p* < 0.05).

Data from the video-clip stimulus evaluation and personality trait assessment: The CATEGORIZATION of emotions triggered by provocative aggressive interactions (i.e., video-clips) correlated negatively (*r* = −0.91) between self-reported ANGER and FEAR ratings on an 11-point scale (see above). ANGER correlated positively with approach decisions (*r* = 0.56), while it correlated negatively with the decision to withdraw from the social interaction (*r* = −0.62). FEAR correlated negatively with the approach decisions (*r* = −0.51) and with the decision to remain passive as well (*r* = −0.53). Self-reported FEAR also correlated positively with the decision to withdraw (*r* = 0.71). Further, self-reported ANGER correlated positively, and self-reported FEAR correlated negatively with personality traits that indicated the proneness to spontaneous (proactive) aggression (ANGER: *r* = 0.43; FEAR: *r* = −0.38) and proneness to reactive aggression (ANGER: *r* = 0.56; FEAR: *r* = −0.42), and it also correlated with the sum-score of the proneness to aggressive behavior (ANGER: *r* = 0.47; FEAR: *r* = −0.39) measured through the FAF (Hampel and Selg, 1975). Self-reported ANGER and FEAR also correlated (ANGER: *r* = 0.59; FEAR: *r* = −0.50) with the physical aggression scale of the AQ (Aggression Questionnaire; Buss and Perry, 1992).

Data from the sentence stimulus evaluation and personality trait assessment: The same pattern of correlations between FEAR and ANGER CATEGORIZATION values and personality trait scores as reported above for the video-clips was found for the negative sentences. Self-reported ANGER and FEAR ratings on an 11-point scale (see above) correlated with trait spontaneous (proactive) aggression (ANGER: *r* =0.60; FEAR: *r* = −0.61), reactive aggression (ANGER: *r* = 0.49; FEAR: *r* = −0.45), and the sum-score of aggression of the FAF (see above; ANGER: *r* = 0.45; FEAR: *r* = −0.45) and with ratings on the physical aggression scale (ANGER: *r* = 0.54; FEAR: *r* = −0.46) of the AQ (Buss and Perry, 1992) as well. Additionally, self-reported ANGER correlated positively with verbal aggression ratings on the AQ (Buss and Perry, 1992; *r* = 0.38).

Data from the experimental decision making runs (i.e., preference index PI) and personality trait assessment: Scores of the PI correlated negatively with the inhibition factor in the FAF in case of neutral (CC neu-A: *r* = −0.65) and negative (CC neg-A: *r* = −0.43) sentences preceding provocative aggressive interactions (see CCs neu-A and neg-A as illustrated in Figure 1A). Further, the PI correlated positively with physical aggression ratings on the AQ (Buss and Perry, 1992; CC neu-A: *r* = 0.51; CC neg-A: *r* = 0.47). Furthermore, PI scores for neutral social interactions (displayed in the video-clips) preceded by negative sentences (CC neg-N, see Figure 1A) also correlated negatively with the inhibition factor of the FAF (Hampel and Selg, 1975; *r* = −0.44) and positively with physical aggression ratings on the AQ (Buss and Perry, 1992; *r* = 0.53).

In order to inspire future research, several dot-biserial correlations were calculated between GENDER (male = 1; female = 2), several personality traits, and decisions. For both negative sentences (NEG, see Figure 1A) and provocative aggressive interaction video-clips (A, see Figure 1A), ANGER ratings were associated with males (NEG-sentences: *r* = −0.43; A-video-clips: *r* = −0.55). FEAR ratings were related to females (NEG-sentences: *r* = 0.4; A-video-clips *r* = 0.48). Familiarity with provocative aggressive interactions presented in video-clips was related to males (A-video-clips: *r* = −0.38). For neutral interaction video-clips (N, see Figure 1A), FEAR ratings were associated with females (*r* = 0.45) and serenity ratings were associated with males (*r* = −0.39).

Based on the evaluation data, in provocative aggressive interactions (A-video-clips, see Figure 1A), approach decisions were related to males (*r* = −0.52), while withdrawal decisions were associated with females (*r* = 0.48). Based on data from the experimental runs (i.e., PI values), provocative aggressive interactions (A-video-clips, see Figure 1A) were related to lower approach decisions in females in case of preceding neutral (CC NEU-A: *r* = −0.58) and negative sentences (CC NEG-A; *r* = −0.57). Neutral interaction video-clips (N-video-clips, see Figure 1A) were related to fewer approach decisions (PI values) in females when preceded by N-sentences (*r* = −0.51).

The sum score of the FAF (Hampel and Selg, 1975) was related to males (*r* = −0.37), and in particular the reactive aggression score (*r* = −0.38). The subscale inhibition of aggression of the FAF (Hampel and Selg, 1975) was related to female gender (*r* = 0.54). Scores of the AQ (Buss and Perry, 1992) on the physical aggression subscale were related to males (*r* = −0.78). Both the SFT (Von Zerssen, 1994) and NEO-FFI (Costa and McCrae, 1992) subscales “openness to experience” were related to males (SFT: *r* = −0.51; NEO-FFI: *r* = −0.41).

## Discussion

We examined if sentences preceding video-clips displaying social interactions affected decisions on these interactions. The sentences provided information about social contexts that was relevant to the social interactions. The video-clips were filmed from a first-person perspective in order to ensure that participants got deeply involved into the social interactions. The experimental material (sentences and video-clips) was constructed in a way that it presented realistic social interactions. The material was validated on different dimensions (e.g., arousal, valence, unambiguity, and emotional category). The sentences (= context attribute 1) preceding the social interactions displayed in video-clips (= context attribute 2) modulated decisions on these social interactions. Note that the social interactions were of different valence and emotional category (i.e., neutral, socially positive, and provocative aggressive). Results are discussed in detail in the following sections.

### Evaluation of the Experimental Material (Sentences and Video-Clips)

We predicted that our experimental material (sentences providing social information and video-clips displaying social interactions) reliably produced similar or even the same patterns of findings as Fehr et al. (2014). Our results supported all predictions. Both negative sentences and the video-clips showing provocative aggressive social interactions received highest arousal ratings followed by arousal ratings of socially positive interactions displayed in video-clips. Finally, the, lowest arousal ratings were observed on neutral social interactions (cf., Bradley and Lang, 1994). How intense participants assessed that the sentences reflected the same differences between the categorization of emotions felt in response to the social interaction watched in video-clips. Further, the arousal and the valence ratings of the preceding sentences confirmed our categorization into phrases of negative, neutral, or positive social contents.

As predicted, both reading sentences with a negative social content and watching video-clips with provocative aggressive interactions mainly produced feelings of anger and fear (cf., Ramirez and Andreu, 2006). In contrast, socially positive interactions resulted more often in feelings of happiness than any other social interaction. Neutral interactions mostly led to feelings of serenity.

The social interactions shown in the video-clips were also rated for familiarity and unambiguity. Participants rated provocative aggressive interactions lowest on familiarity, followed by ratings of neutral interactions. Socially positive interactions were associated with the highest familiarity ratings. The clarity of understanding what was going on in a given social interaction was assessed on an unambiguity rating scale. Distribution of scores on this scale indicated that most social interactions were understandable and clear to participants.

Summarizing, the assessments both of the sentences supposed to create certain social contexts prior to the presentation of the video-clips and the assessments of the video-clips themselves largely confirmed the predictions inferred from Fehr et al. (2014). The experimental materials could therefore be used for exploring peoples' decisions on social interactions in a certain social context in future research.

### Modulation of Decisions on Social Interactions: The Utility of Combining Information From Two Sources (Sentences and Video-Clips)

One of the main goals of the present study was to explore the impact of information about a context (provided in a sentence = context attribute 1) prior to watching a social interaction (i.e., a short video-clip displaying a social interaction filmed from a first-person view = context attribute 2) on approach or withdrawal decisions. We suggested that the information provided in the sentences and in the video-clips were integrated and used jointly for decisions in different emotional social contexts (i.e., in seven different sentence-video-combinations labeled as experimental Context Conditions = CCs). An uncertainty index revealed that participants consistently decided on how to respond to a given social interaction across two experimental runs.

In line with Bettencourt et al. (2006), who suggested that provocative information (e.g., the presence of a gun) might facilitate aggressive actions, one might assume that negative contextual information raises the chance to respond with situationally adequate behavior if this information fits to the contexts. That is, additional contextual information, in case of our research provided by the sentences, can increase the probability to act reactively aggressive to defend oneself in a provocative aggressive (i.e., proximally threatening) interaction or to act prosocially in a positive interaction. Our data confirmed this assumption and showed that provocative aggressive interaction scenarios that were preceded by sentences that contained negative social information (e.g., “Tom just stole your mothers handbag”) resulted more often in the decision to respond in a reactively aggressive manner to this situation (i.e., participants choose the approach option) than in the same provocative aggressive interaction that was preceded by a neutral sentence. Positive interactions (i.e., friendly interactions) that were preceded by positive information resulted more often in prosocial approach decisions in comparison to the same positive interaction that was preceded by neutral information provided in a sentence, that the sentences impacted the decisions on how to respond to a social interaction differently, depending on their content. For instance, neutral social interactions that followed sentences with negative social content ended up in less approach decisions. In contrast, the same neutral social interactions preceded by positive social contents led to more approach decisions than being preceded by neutral social contents.

Summarizing, context information that was displayed prior to social interactions modulated decisions on how to respond to a given social interaction. This observation confirmed assumptions about the modulatory potential of context information in quasi-realistic decision situations (cf., Klein, 2008), even in a laboratory context. Future research should elaborate on this quasi-realistic experimental laboratory concept in order to investigate different emotional dimensions in different types of complex social contexts and their impact on decisions. Our experimental material could be used to investigate decisions in social interactions framed by different social contexts in diverse and subgroups of people, for instance, in varying age cohorts, individuals with different violence socialization, and different genders [see also Anderson and Bushman (2002)].

### Relationships Between Aggression- and Violence-Related Traits, Stimulus Evaluation Scores, and Social Decision Making

As predicted, anger as a response to social interactions presented in video-clips correlated with approach decisions (i.e., the decision to show reactive aggression or to start fighting in order to defend oneself). Fear as an emotional response on provocative aggressive interactions correlated with preferences for withdrawal (i.e., flight from proximal threat; cf., Strüber and Fehr, 2009). In line with these findings, traits related to aggression [i.e., physical, proactive, reactive, and the sum score of aggression; see Hampel and Selg (1975) and Buss and Perry (1992)] correlated positively with anger and negatively with fear (cf., Ramirez and Andreu, 2006). The preference index (PI = the tendency to approach a situation and to get actively involved in it) in trials, in which negative social information was provided prior to provocative aggressive video-clips, correlated negatively with the trait “inhibition of aggression” and positively with the trait “physical aggression.” In short, correlations showed significant relationships between provocative aggressive or violent contexts, personality traits, and social decisions. The correlations hence validated our quasi-realistic experimental approach to investigate peoples' decisions in social interactions of a different nature (e.g., provocative aggressive, or friendly) in a laboratory environment.

In line with previous literature, our exploratory correlation analyses confirmed that males tended to report anger more often than females in threatening social interactions (cf., Bettencourt and Miller, 1996). Females reported to feel fear more often than males if a sentence provided negative social information, and a video-clip displayed a provocative aggressive social interaction (cf., Bettencourt and Miller, 1996; Strüber and Fehr, 2009). Males also reacted more often aggressively by choosing the approach option in provocative aggressive interactions, while females did less (cf., Bettencourt and Miller, 1996; Archer, 2004; Wahl, 2009). Females rather preferred to withdraw from those provocative aggressive interactions. In accordance with these gender differences on how to respond to provocative aggressive interactions, males showed higher scores on reactive and physical aggression and lower scores on aggression inhibition traits than females [see Hampel and Selg (1975) and Buss and Perry (1992)]. Males also rated their familiarity with provocative aggressive interactions higher than females [see also Hyde (2014)]. Despite these significant gender differences that confirmed prior literature on aggression (cf. Archer, 2004; Strüber and Fehr, 2009; Wahl, 2009; Hyde, 2014), these correlations should be interpreted cautiously as our subsample sizes on genders were rather small.

### Take-Home Message

Our findings revealed that if a social interaction displayed in a quasi-naturalistic video-clip was prosocial, positive preceding information (e.g., a social content like “It's always a pleasure to meet with Simon.”) significantly increased the likelihood of approaching the social interaction. In contrast, approach decisions in provoking aggressive social interactions (i.e., self-defense responses) were supported by preceding negative information. Decisions in neutral social interactions interacted with preceding positive information resulting in more prosocial involvement decisions (i.e., approach decisions), while negative preceding information increased the likelihood of withdrawal decisions, and neutral preceding information led to both proactive and withdrawal decisions.

The video-clips were evaluated reliably (cf., Fehr et al., 2014) and can therefore be used validly in future studies. Our quasi-realistic, experimental multi-attribute approach (i.e., sentences and videos) combined information provided prior to watching a real-life social interaction and indicated that information that occurs prior to social interactions can influence social decisions to be made in these situations. Hence, we recommend that the quasi-realistic experimental framework used in the present paper should be developed further for studying how stimuli related to real-life contexts are cognitively processed and how they affect social decision making (cf., Miedl et al., 2010; Doehring, 2016; Gloy et al., 2020).

## Data Availability Statement

The raw data supporting the conclusions of this article will be made available by the authors, without undue reservation.

## Ethics Statement

Ethical approval was not provided for this study on human participants because at Bremen University, behavioral studies involving human individuals do not require an approval by the ethics committee. The patients/participants provided their written informed consent to participate in this study.

## Author Contributions

TF: conceptualization, methodology, software, formal analysis, visualization, investigation, and writing—original draft. AA: conceptualization, methodology, writing—review and editing, and project administration. Both authors contributed to the article and approved the submitted version.

## Conflict of Interest

The authors declare that the research was conducted in the absence of any commercial or financial relationships that could be construed as a potential conflict of interest.

## Publisher's Note

All claims expressed in this article are solely those of the authors and do not necessarily represent those of their affiliated organizations, or those of the publisher, the editors and the reviewers. Any product that may be evaluated in this article, or claim that may be made by its manufacturer, is not guaranteed or endorsed by the publisher.
